# The effects of type 2 diabetes mellitus on postural adaptation and complexity during a visual search task in older adults

**DOI:** 10.3389/fnagi.2025.1650484

**Published:** 2025-10-23

**Authors:** Mahdiyeh M. Manafi, Alexander Wolfe, Junhong Zhou, Wanting Yu, Ikechukwu Iloputaife, Tongjian You, Brad Manor, Azizah J. Jor’dan

**Affiliations:** ^1^Department of Exercise and Health Sciences, University of Massachusetts Boston, Boston, MA, United States; ^2^Hinda and Arthur Marcus Institute for Aging Research, Hebrew SeniorLife, Boston, MA, United States; ^3^Harvard Medical School, Boston, MA, United States

**Keywords:** attention, diabetes mellitus, type 2, entropy, postural balance, task performance

## Abstract

**Background:**

Type 2 diabetes mellitus (T2DM) negatively impacts both the peripheral and central systems involved in postural control, leading to impairments, especially under dual-task conditions. This study investigated the effects of T2DM on postural sway using single- and multi-scale metrics during an attention-demanding dual-task standing protocol.

**Methods:**

Twenty-four relatively healthy older adults (76 ± 6 years) and 20 older adults with T2DM (76 ± 7 years) completed quiet standing (i.e., single-task, control) and dual-task (i.e., visual search) conditions. For the dual-task condition, participants counted the frequency of a designated letter in a block of text. Postural sway (i.e., elliptical area, jerk, path length, and range of acceleration) was assessed using a wearable motion sensor. Multi-scale entropy was used to quantify the complexity of postural sway in the medial-lateral (ML) and anterior-posterior (AP) directions. Postural adaptation was calculated as the percent change in sway metrics from control to visual search condition. Task performance was measured as percent accuracy in the visual search task.

**Results:**

Compared to the non-diabetic group, the T2DM group exhibited greater elliptical sway area (*p* = 0.007), jerk (*p* = 0.001), path length (*p* < 0.0001), and range of acceleration (*p* = 0.006), and lower ML sway complexity (*p* = 0.053), irrespective of task condition. There were no group differences in postural adaptation for any sway metric. Across participants, single-scale sway metrics were lower during the visual search compared to the control condition (*p* < 0.05). Within the non-diabetic group only, visual search performance was correlated with postural adaptation in elliptical sway area (*r* = −0.70, *p* < 0.0001) and range of acceleration (*r* = −0.66, *p* = 0.0009).

**Conclusion:**

The T2DM group exhibited altered single-scale sway measures and reduced ML sway complexity, highlighting the sensitivity of both single- and multi-scale postural sway metrics in detecting group differences in standing postural control. The link between postural adaptation and visual search performance, which was evident only in the non-diabetic group, may suggest a decoupling between perception and action in patients with T2DM.

## Introduction

Maintaining balance is essential for everyday activities. In many real-world situations, individuals engage in cognitive tasks–such as reading, talking, or problem-solving–while standing. Simultaneously performing standing postural control and cognitive tasks (i.e., dual-tasking) often leads to a decline in cognitive performance, the postural response, or both (Boisgontier et al., 2013). A healthy postural control system adjusts to such dual-tasking through a process known as postural adaptation (Haddad et al., 2013). Interestingly, the nature of the cognitive task may influence how this adaptation occurs. For example, tasks that involve visual search may benefit from reduced postural sway to enhance visual stability, whereas tasks like mental arithmetic (e.g., counting backward) may not require such adjustments (Stoffregen et al., 2000, 2007). These task-related changes in posture–reflected as differences between single-task and dual-task conditions–are referred to as postural adaptation and reflect the body’s attempt to optimize performance under increased cognitive load. Aging and age-related conditions, such as type 2 diabetes mellitus (T2DM), oftentimes diminish balance control (Gorniak et al., 2019), resulting in increased risk of falls (Morrison et al., 2012). The maintenance of standing balance depends upon a complex regulatory system that involves the integration of sensory information from the visual, somatosensory, and vestibular systems (Horak, 2006).

Standing postural control can be traditionally characterized by examining the characteristics of postural sway of the body, as measured by center-of-pressure (COP) fluctuations. These measurements can be derived from a single scale of time (e.g., area, path length) or more recent multi-scale metrics, such as multi-scale entropy (MSE), which are designed to capture the information content or “complexity” of the signal (Peterka, 2002). Prior research has demonstrated that visual search tasks can influence the temporal dynamics of sway in older adults (Jor’dan et al., 2015; Koslucher et al., 2012), underscoring the sensitivity of postural control to concurrent cognitive demands. Consistent with this line of work, our prior study supported the notion that, in addition to traditional metrics, postural sway complexity during a visual search task may serve as a sensitive marker for detecting postural control changes in older adults with cognitive impairment [i.e., Alzheimer’s disease, mild cognitive impairment (MCI)] (Zhou et al., 2023). However, the impact of performing a visual search task on standing postural sway dynamics in older adults with T2DM has not been investigated.

Aging complicated by T2DM disrupts the integration of multiple sensory and motor inputs that operate across different temporal scales (Mahoney et al., 2021). This disruption degrades the physiological feedback loops that underpin both postural adaptation and complexity during cognitively demanding tasks such as visual search (Manor and Lipsitz, 2013; Manor et al., 2010). Addressing this gap is critical for identifying the mechanisms through which T2DM impairs balance and for informing targeted interventions to manage or improve dual-task standing balance within this population. We thus measured single- and multi-scale measures of standing postural sway during the performance of an attention-demanding visual search dual-task protocol in older adults with and without T2DM. We hypothesized that (1) compared to non-diabetics, those with T2DM would exhibit reduced postural stability, reflected in worse single- and multi-scale sway metrics (i.e., greater elliptical sway area, jerk, path length and range, as well as lower sway complexity) across both control and dual-task conditions; (2) Across all participants, postural sway would be worse during the dual-task visual search compared to the control condition; (3) Older adults with T2DM would exhibit worse postural adaptation (i.e., a larger percentage change in each postural sway metric from the control to the visual search condition) compared to their non-diabetic counterparts; and (4) Participants who exhibited less adaptive postural responses during the dual-task visual search condition would exhibit poorer visual search task performance.

## Materials and methods

### Participants

This analysis was derived from a cross-sectional parent study of 44 participants: 24 non-diabetic older adults and 20 older adults with T2DM. Participants for the parent study were recruited through local community and online advertisements, an institutional research participant repository, and convenience sampling. An *a priori* power analysis was calculated based on a previous study by Lipsitz et al. (2000), which included 20 participants (10 young, 10 older adults). In that study, older adults demonstrated a lower change in cerebral blood flow velocity from sitting to standing compared to young adults (−15.2 ± 3.1 cm/s versus −18.8 ± 1.5; effect size of 1.5). This analysis indicated that a minimum sample size of 26 (13 per group) would provide ≥90% power to detect similar between-group differences at an alpha level of 0.05.

Inclusion criteria for both groups were as follows: aged 65 years or older with no cognitive impairment as determined by a Mini-Mental State Exam (MMSE) score of ≥27. Those with a physician diagnosis of type 2 diabetes mellitus were included in the T2DM group. The exclusion criteria included any acute medical conditions requiring hospitalization within the past 6 months; self-reported insulin-dependent diabetes; self-reported diabetic foot ulcers or severe diabetic neuropathy; self-reported history of cardiopulmonary disease, neurological disease, or metabolic disease; pain or musculoskeletal disorder that significantly influences postural control; history of stroke; severe visual impairment; uncontrolled hypertension; current recreational drug or alcohol abuse; inability to stand continuously for at least 5 min without personal assistance; or the inability to understand and communicate in English. All participants provided written informed consent as approved by the Institutional Review Board of Hebrew Senior Life.

### Study protocol

#### Instrumentation

Participants were instrumented with the Mobility Lab, a validated and reliable wearable inertia sensor-based device for measuring gait and balance (APDM Wearable Technologies, Portland, OR) (Morris et al., 2019). Standing postural sway was recorded using the lumbar sensor placed on the lower back of each participant.

### Postural control assessment protocol

A screen was projected 3 meters in front of each participant at eye level. Participants completed two trials of quiet standing (i.e., single-task, control) and dual-task standing (i.e., visual search) (Chang et al., 2010; Jor’dan et al., 2015; Koslucher et al., 2012). For the control condition, participants were instructed to stand comfortably with their arms down at their sides and maintain their gaze within the boundaries of a blank white screen. For the visual search dual-task condition, participants were instructed to count the frequency of one target letter (i.e., T or P, one for each of the two trials) in a panel of 156 black randomized letters (serif typeface: Times New Roman; size: 32 pt; standard-sized spacing) displayed on a blank white screen of the same dimensions as the control condition. If participants completed the visual search before the end of the 60-s trial, they were instructed to go back and “check their count.” At the end of each trial, participants reported their count of the designated letter and where their count stopped in the grid.

### Data analysis

#### Postural sway metrics

Single-scale postural sway measures were elliptical sway area (m^2^/s^4^), jerk (m^2^/s^5^), path length (m/s^2^), and range of acceleration (m/s^2^), which are measures used to assess fall risk in older adults (Howcroft et al., 2017; Huang and Brown, 2013).

For the COP time-series, we employed MATLAB (r2022a; MathWorks, Natick, MA, USA) to implement empirical mode decomposition (EMD) for the purpose of eliminating fluctuations above 20 Hz and below 0.2 Hz. MSE analysis was used to quantify the complexity of the postural sway time-series signal for the medial-lateral (ML) and anterior-posterior (AP) direction. To achieve this, we applied well-established parameters that have been previously documented, ensuring the elimination of physiologically unnecessary processes while identifying the appropriate number of dynamic patterns within the approximation (Zhou et al., 2017). Following the filtration of these time series, we performed a “coarse-graining” process to capture the system dynamics. The COP time-series was divided into overlapping windows of a specific length, determined by a scale factor (τ) ranging from 1 to 8 data points. To calculate the sample entropy of each coarse-grained time-series, we adopted recommendations from previous studies, setting the parameters as *m* = 2 and *r* = 15% (Gow et al., 2015; Wu et al., 2013). Subsequently, we generated an MSE curve based on fluctuations. The metric for postural sway complexity was calculated by determining the area under the MSE curve. In this context, smaller areas denote lower sample entropy values across multiple time scales, indicating lower complexity.

Single- and multi-scale postural sway means were determined per condition (control, visual search) and averaged across like trials.

#### Postural adaptation during performance of visual search task

Postural adaptation in response to the dual-task was quantified as the percentage change in postural sway metrics (i.e., elliptical sway area, jerk, path length, range of acceleration, as well as ML and AP complexity) from the control to the visual search condition using the following formula:


$$\left(\mathrm{sway}\,{\mathrm{metric}}_{\mathrm{visual}\,\mathrm{search}}-\mathrm{sway}\,{\mathrm{metric}}_{\mathrm{control}}\right)/\left({\mathrm{swaymetric}}_{\mathrm{control}}\right)\times 100$$

#### Visual search task performance

Task performance was calculated as the percent accuracy (i.e., the number of target letters reported by the participant divided by the total number of target letters within the amount of letters scanned, multiplied by 100).

### Statistical analyses

Descriptive statistics were used to summarize participant characteristics. Outcomes are expressed as mean ± SD. Student’s *t*-test or Fisher’s Exact test were used to compare these characteristics between groups (i.e., non-diabetic, T2DM). All outcomes of postural sway were normally distributed.

A repeated-measures analysis of variance (rANOVA) was conducted to test our hypotheses regarding group differences and task effects. We hypothesized that, compared to non-diabetic older adults, the T2DM group would exhibit worse single- and multi-scale postural sway, regardless of task condition, with this deterioration being more pronounced during the visual search condition. The dependent variables were elliptical sway area, jerk, path length, range of acceleration, ML sway complexity, or AP sway complexity during the control or visual search conditions. Model effects were group (non-diabetic, T2DM), condition (control, visual search), and their interaction. Separate models were used for each dependent variable. All models were adjusted for age, sex, and hypertension.

We used one-way ANOVA models to test adaptation differences. We hypothesized that the T2DM group would exhibit worse postural adaptation (i.e., positive percentage change in each single-scale and multi-scale postural sway metric from the control to the visual search condition) compared to non-diabetic older adults. This reflects an impaired ability to reduce postural motion during performance of a visual search task. The dependent variables for the postural sway metrics were the postural adaptation as measured by elliptical sway area, jerk, path length, range of acceleration, and ML or AP sway complexity. The model effect was group (non-diabetic, T2DM). Separate models were conducted for each dependent variable. Models were adjusted for age, sex, and hypertension.

We used linear regression models to examine the relationship between posture sway and cognition. We hypothesize that dual-task visual search postural sway metrics (i.e., single-scale and multi-scale) and their postural adaptation in response to dual-task would correlate with visual search task performance (i.e., percent accuracy). Models were adjusted for age, sex, and hypertension.

To control for family-wise error due to multiple comparisons, we applied the Bonferroni correction within outcome families. For single-scale sway metrics (elliptical sway area, jerk, path length, range of acceleration; 4 tests), the adjusted alpha level was set to 0.0125 (0.05 ÷ 4). For multi-scale measures (ML and AP complexity; 2 tests), the alpha level was set to 0.025 (0.05 ÷ 2).

The partial eta squared (η^2^) was used to examine the effect size of the ANOVA results, and 0.01 ≤ η^2^ < 0.06, 0.06 ≤ η^2^ < 0.14, and η^2^ ≥ 0.14 were considered small, medium, and large effect sizes, respectively (Cohen, 2013). Analyses were performed using JMP Software (SAS Institute, Cary, NC, United States).

#### Exploratory analysis

Prior research indicates reliable sex differences in postural sway and falls risk among older adults (Era et al., 2006; Ishimoto et al., 2009; Kim et al., 2010; Stevens and Sogolow, 2005). These findings suggest that sex influences postural control and thus warrants consideration in studies examining diabetes-related changes. We conducted a rANOVA to test the effect of sex differences on single-scale and multi-scale postural sway metrics. The dependent variables were elliptical sway area, jerk, path length, range of acceleration, ML sway complexity, or AP sway complexity during the control or visual search conditions. Model effects were sex (male, female), group (non-diabetic, T2DM), condition (control, visual search), and their interaction. Separate models were used for each dependent variable. All models were adjusted for age and hypertension.

## Results

### Participant characteristics

Forty-four participants (non-diabetic = 24; T2DM = 20) were included in the analyses. Groups were similar in age, sex distribution, BMI, years of education, hypertension status, global cognition (i.e., MMSE scores), and visual performance accuracy (*p* > 0.25) (Table 1). All participants reported being able to clearly see the visual search letters presented during the task. All postural sway outcomes were normally distributed (Table 2).

**TABLE 1:** Group demographics and clinical characteristics.

Characteristics	Non-diabetic	T2DM	*P-value*
N	24	20	
Age (years)	76 ± 6	76 ± 7	0.87
% female	50	60	0.76
BMI (kg/m^2^)	29 ± 5	30 ± 6	0.47
Education (years)	17 ± 3	16 ± 2	0.73
Hypertension (% yes)	54	63	0.75
MMSE score	29 ± 1.2	28 ± 1	0.25
Visual search accuracy (%)	88 ± 1	89 ± 10	0.85

MMSE, Mini-Mental State Exam; Data = means ± SD.

**TABLE 2:** Postural sway metrics by group.

Postural sway metrics	Non-diabetic	T2DM	*P*-value
**Single-scale metrics**			
**Control**			
Elliptical sway area (m^2^/s^4^)	0.07 ± 0.05	0.11 ± 0.09	0.08
Jerk (m^2^/s^5^)	0.38 ± 0.24	0.81 ± 0.72	0.01*
Path length (m/s^2^)	12.66 ± 3.27	18.17 ± 7.80	0.003*
Range of acceleration (m/s^2^)	0.69 ± 0.25	0.82 ± 0.36	0.17
**Visual search**			
Elliptical sway area (m^2^/s^4^)	0.04 ± 0.03	0.08 ± 0.07	0.01*
Jerk (m^2^/s^5^)	0.21 ± 0.12	0.39 ± 0.29	0.01*
Path length (m/s^2^)	10.07 ± 2.55	13.60 ± 4.60	0.003*
Range of acceleration (m/s^2^)	0.50 ± 0.16	0.72 ± 0.30	0.004*
**Multi-scale metrics**			
**Control**			
ML complexity	1.84 ± 0.15	1.75 ± 0.21	0.11
AP complexity	1.50 ± 0.20	1.50 ± 0.19	0.91
**Visual search**			
ML complexity	1.87 ± 0.15	1.79 ± 0.19	0.13
AP complexity	1.57 ± 0.18	1.56 ± 0.14	0.82

Data = mean ± SD; *p*-values were from unadjusted ANOVA models *P-value < 0.0125.

### The effect of type 2 diabetes and task condition on single-scale postural sway metrics

**FIGURE 1:**
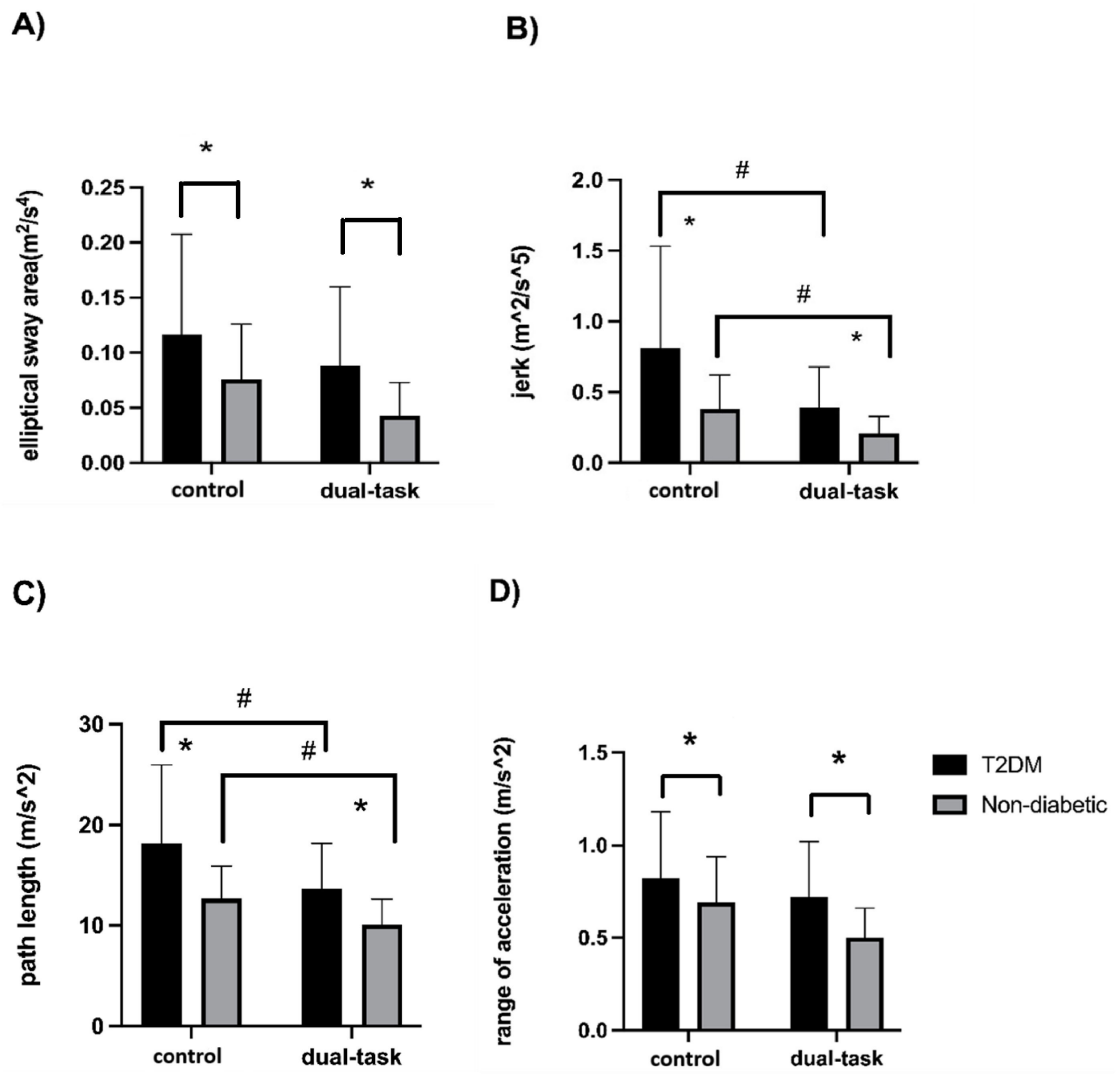
Single-scale measures of elliptical sway area **(A)** jerk **(B)** path length **(C)**, and range of acceleration **(D)** by group during the control (i.e., quiet standing) and visual search (i.e., dual-task) condition. Compared to non-diabetic, the T2DM group displayed greater elliptical sway area (*p* = 0.007), jerk (*p* = 0.001), path length (*p* < 0.0001), and range of acceleration (*p* = 0.006), irrespective of task condition. Across participants, jerk (*p* = 0.002) and path length (*p* = 0.002) were lower during the performance of the visual search compared to control condition independent of age, sex, and hypertension. *Denotes a significant difference between the T2DM and non-diabetic groups; #Denotes a significant difference between single-task and dual-task conditions.

Repeated measures ANOVA models, adjusted for age, sex, and hypertension, revealed a main effect of group for all single-scale measures and a main effect of task condition for two measures (i.e., jerk and path length). Compared to the non-diabetic group, the T2DM group had greater elliptical sway area [*F*(1,36) = 7.45, *p* = 0.007, ηp^2^ = 0.09; Figure 1A], jerk [*F*(1,36) = 11.40, *p* = 0.001, ηp^2^ = 0.13; Figure 1B], path length [*F*(1,37) = 18.64, *p* < 0.0001, ηp^2^ = 0.19; Figure 1C], and range of acceleration [*F*(1,37) = 7.95, *p* = 0.006, ηp^2^ = 0.09; Figure 1D] irrespective of task condition.

Across participants, the magnitude of jerk [*F*(1,36) = 10.36, *p* = 0.002, ηp^2^ = 0.12; Figure 1B] and path length [*F*(1,37) = 10.84, *p* = 0.002, ηp^2^ = 0.12; Figure 1C] was lower during the performance of the visual search condition compared to the control condition, independent of age, sex, and hypertension. The elliptical sway area [*F*(1,36) = 4.90, *p* = 0.029, ηp^2^ = 0.06; Figure 1A] and range of acceleration [*F*(1,37) = 5.75, *p* = 0.019, ηp^2^ = 0.07; Figure 1D] were slightly lower during the visual search dual-task condition compared to the control condition. However, these effects did not reach statistical significance after Bonferroni correction, though they demonstrated a trend toward reduced sway during dual tasking. No group × condition interaction was observed for any of the sway metrics (*F* < 2.02, *p* > 0.15).

### The effect of type 2 diabetes and task condition on multi-scale postural sway complexity

**FIGURE 2:**
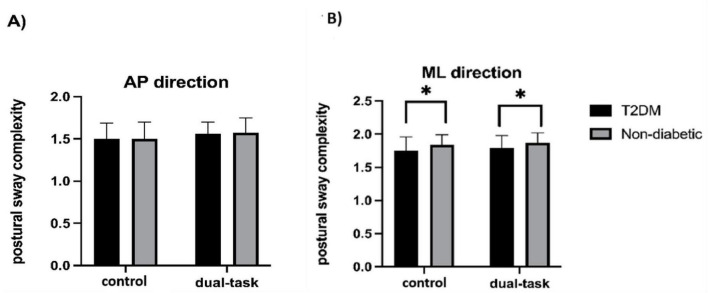
Postural sway complexity in the anterior-posterior (AP) **(A)** and medial-lateral (ML) **(B)** directions by group during the control (i.e., quiet standing) and visual search (i.e., dual-task) condition. The T2DM displayed lower ML sway complexity compared to the non-diabetic group irrespective of task condition (Unadjusted: *p* = 0.02). After controlling for age, sex, and hypertension, the group effect on ML sway complexity trended toward significance (*p* = 0.05). *Denotes a significant difference between the T2DM and non-diabetic groups.

The unadjusted rANOVA model revealed significant main effects of group on ML sway complexity [*F*(1,38) = 5.20, *p* = 0.025, ηp^2^ = 0.06; Figure 2B], but not in AP sway complexity [*F*(1,38) = 0.004, *p* = 0.952, ηp^2^ = 0.00004; Figure 2A]. Compared to the non-diabetic group, the T2DM group exhibited significantly lower ML postural sway complexity irrespective of task condition (Figure 2B). After controlling for age, sex, and hypertension, however, the group effect on ML sway complexity was no longer significant [*F*(1,38) = 3.85, *p* = 0.053, ηp^2^ = 0.045].

For both ML and AP sway complexity, there were no main effects of condition [ML: *F*(1,38) = 0.72, *p* = 0.40, ηp^2^ = 0.009; AP: *F*(1,38) = 2.36, *p* = 0.129, ηp^2^ = 0.027], nor were there group × condition interactions [ML: *F*(1,84) = 0.01, *p* = 0.91, ηp^2^ = 0.0002; AP: *F*(1,38) = 0.05, *p* = 0.827, ηp^2^ = 0.0006].

### The effect of type 2 diabetes on postural adaptation

One-way ANOVA analysis revealed no main effect of group on postural adaptation during visual search for elliptical sway area [*F*(1, 38) = 1.39, *p* = 0.245, ηp^2^ = 0.035], jerk [*F*(1, 38) = 0.12, *p* = 0.731, ηp^2^ = 0.003], path length [*F*(1, 38) = 0.04, *p* = 0.848, ηp^2^ = 0.001], range of acceleration [*F*(1, 38) = 0.75, *p* = 0.391, ηp^2^ = 0.019], or ML and AP sway complexity [*F*(1, 38) = 0.54, *p* = 0.469, ηp^2^ = 0.014; *F*(1, 38) = 0.20, *p* = 0.660, ηp^2^ = 0.005, respectively], after controlling for age, sex, and hypertension.

### The association between postural sway metrics and visual search task performance

**FIGURE 3:**
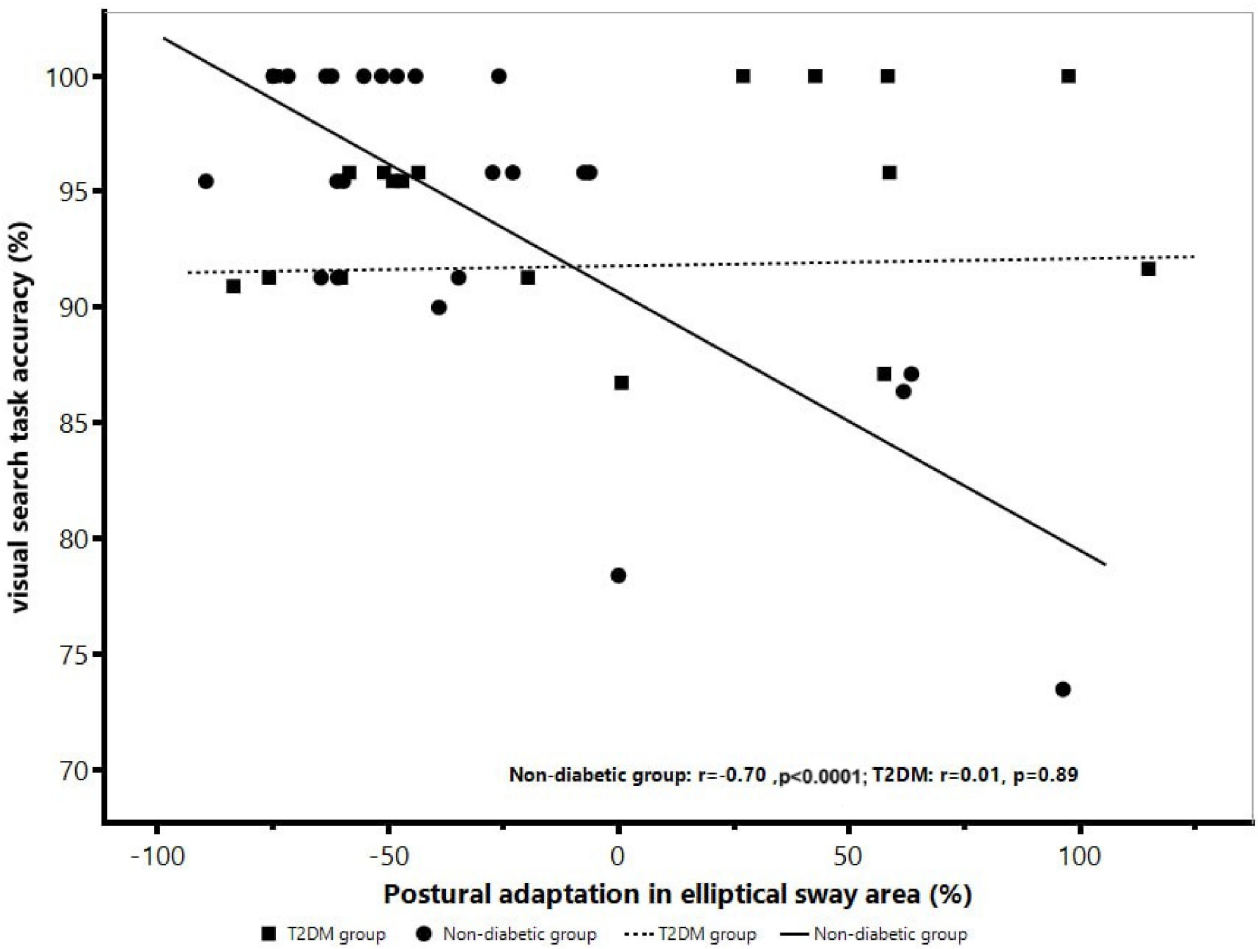
The association between visual search performance and postural adaptation in elliptical sway area within the non-diabetic group and T2DM group. Within the non-diabetic group only, those with greater percent increase in elliptical sway area demonstrated lower visual search task accuracy independent of age, sex, and hypertension (*r* = –0.70, *p* < 0.0001). However, this association was not present in the T2DM group (*r* = 0.01, *p* = 0.89).

Within-group analysis showed a negative association between visual search performance and the postural adaptation in elliptical sway area (*r* = −0.70, *p* < 0.0001; Figure 3) and range of acceleration (*r* = −0.66, *p* = 0.001) within the non-diabetic group only, such that those with worse visual search accuracy displayed greater increase in elliptical sway area and range of acceleration independent of age, sex, and hypertension. This association was not present within the T2DM group (*r* = 0.01, *p* = 0.89) (Figure 3).

There were no within-group associations between visual search accuracy and postural sway complexity in the non-diabetic group (ML: *r* = −0.10, *p* = 0.59; AP: *r* = 0.05, *p* = 0.78) or in the diabetic group (ML: *r* = −0.10, *p* = 0.64; AP: *r* = 0.25, *p* = 0.24).

Similarly, there were no within-group associations between visual search accuracy and postural adaptation to sway complexity during the visual search task in the non-diabetic group (ML: *r* = −0.20, *p* = 0.31; AP: *r* = 0.05, *p* = 0.80) or in the diabetic group (ML: *r* = −0.10, *p* = 0.58; AP: *r* = 0.07, *p* = 0.73).

### Exploring sex differences in postural sway metrics

rANOVA revealed no main effect of sex on elliptical sway area [*F*(1, 36) = 2.67, *p* = 0.107, ηp^2^ = 0.034], path length [*F*(1, 37) = 0.19, *p* = 0.665, ηp^2^ = 0.002], jerk [*F*(1, 37) = 0.14, *p* = 0.714, ηp^2^ = 0.002] and range of acceleration *F*(1, 37) = 0.07, *p* = 0.792, ηp^2^ = 0.001. However, rANOVA models, adjusted for age and hypertension, revealed a main effect of sex on ML sway complexity [*F*(1,38) = 18.41, *p* < 0.0001, ηp^2^ = 0.191], but not on AP sway complexity [*F*(1,38) = 2.80, *p* = 0.10, ηp^2^ = 0.035]. Females exhibited lower ML postural sway complexity, compared to males, irrespective of group and condition.

## Discussion

The current study aimed to determine the effect of T2DM on measured single- and multi-scale measures of standing postural sway during the performance of an attention-demanding visual search dual-task protocol in older adults with and without T2DM. Our results indicated that compared to the non-diabetic group, the T2DM group had greater elliptical sway area, jerk, path length, and range of acceleration irrespective of task condition, which is indicative of reduced postural stability. Additionally, the T2DM group exhibited lower ML postural sway complexity regardless of task condition; however, this difference was no longer significant after adjusting for age, sex, and hypertension. There were no group differences in postural adaptation during the visual search task, as measured by single-scale sway metrics and sway complexity. These results largely confirm our hypothesis and indicate that individuals with T2DM exhibit relatively greater disruption of postural control, as reflected in both single- and multi-scale sway metrics.

Older adults with T2DM are at an increased risk of falls, with impaired balance frequently identified as a significant contributing factor (Hewston and Deshpande, 2016). We observed that the T2DM group had significantly greater elliptical sway area, jerk, path length, and range of acceleration compared to the non-diabetic group, irrespective of task condition. Independent research supports these findings, demonstrating that under both control and dual-tasking conditions (i.e., N-back task), single-scale measures of postural sway, such as path length, were notably greater in the diabetic group (Gorniak et al., 2019; Rodrigues et al., 2023). Rodrigues et al. (2023) investigated postural stability in older adults with T2DM by analyzing COP during a saccadic gaze task performed in a parallel foot stance. Their findings indicated that older adults with diabetes, but without peripheral neuropathy, exhibited increased standing postural sway–reflected in greater mean sway amplitude and mean sway velocity–compared to healthy age-matched controls. These findings collectively emphasize a consistent pattern of compromised postural stability in individuals with T2DM, underscoring the importance of incorporating a comprehensive assessment and intervention of balance into their rehabilitation regimen.

Postural sway is known to modulate in response to secondary task demands. Based on prior studies, the visual search task is considered an “external” task which imposes a visual constraint to stabilize (or reduce) participants’ postural sway (Prado et al., 2007). Such reductions in postural sway have been shown to facilitate visual search performance, denoting the perception-action synergy (Jor’dan et al., 2015; Stoffregen et al., 2000). Our results revealed that across both groups, participants on average reduced their postural sway, as measured by jerk and path length, during performance of the visual search dual-task condition compared to the control condition. Although reductions in elliptical sway area and acceleration range did not reach statistical significance, these metrics showed a trend toward decreased sway during dual-tasking. Our results, however, did not support our hypothesis that individuals with T2DM would exhibit a reduced capacity to attenuate postural sway during performance of the visual search dual-task condition compared to non-diabetic older adults. This may, in part, be attributable to the relatively preserved cognitive function within the T2DM group, as indicated by MMSE scores of ≥27. Prior studies have shown that individuals with cognitive impairments tend to display an increased standing postural sway area, such as a larger sway area, when engaged in a cognitive dual-task (e.g., verbalized serial subtraction), in comparison to cognitively intact older adults (Hauer et al., 2003; Manckoundia et al., 2006; Mesbah et al., 2017). Incorporating a more challenging visual search task (e.g., counting the frequency of two target letters) or manipulating the distance of visual targets (e.g., near versus far) may enhance the assessment and its relationship to sensory-motor integration in individuals with T2DM.

Postural adaptation is a mechanism in which the body maintains stability through the integration of sensory information. Postural adaptation relies on the perception-action system, in which the brain and musculoskeletal system synergize to produce postural adjustments during perturbation (Alexander, 1994). This means that any dysfunction in perception (i.e., cognition) and/or action (i.e., motor) can disrupt this synergy. For example, Manor et al. (2010) investigated the effects of chronic sensory impairments on postural sway complexity in older adults during a verbal serial subtraction dual-task. They found that during quiet standing (single-task condition), postural sway complexity was highest in the control group and progressively declined in those with visual, somatosensory, and combined sensory impairments. Moreover, during the dual-task condition, sway complexity decreased even further, indicating a reduced ability of the postural control system to adapt to perturbations or stressors (Manor et al., 2010). These findings are corroborated by independent studies associating reduced postural sway complexity with frailty (Lipsitz, 2002, 2004) and an elevated risk of future falls (Zhou et al., 2017).

Earlier research has consistently demonstrated a strong link between ML sway and the risk of adverse events, such as falls, in older adults and patient populations (Błaszczyk and Orawiec, 2011; Park et al., 2014; Stel et al., 2003; Sun et al., 2019). The current study provided novel evidence that older adults with T2DM had lower ML standing postural sway complexity compared to non-diabetic older adults; however, the group difference was not significant after controlling for age, sex, and hypertension. These covariates appeared to account for the group effects observed in the ML postural sway measured by multi-scale sway metrics. These findings are consistent with those of Mengarelli et al. (2019), who demonstrated the robustness of entropy measures across various input parameters in a study of older adults with T2DM. Their results revealed significant group differences in COP regularity, particularly in the ML direction, between neuropathic and non-neuropathic participants, as determined by motor and sensory nerve conduction velocities. Specifically, individuals with diabetic neuropathy exhibited lower postural sway complexity, suggesting a diminished capacity to generate adaptable postural responses and greater reliance on rigid balance control patterns (Mengarelli et al., 2019). Moreover, alterations in ML complexity are associated with changes in functions related to standing postural control in various vulnerable populations (Etzelmueller et al., 2020). Therefore, it may be beneficial for rehabilitative strategies to assess and target the complexity in the ML direction.

Numerous metrics derived from non-linear dynamics and chaos theory have been used to study the temporospatial characteristics of postural sway, capturing dynamic features of COP signals often missed by traditional linear measure (Błaszczyk and Klonowski, 2001; Collins et al., 1995; Kȩdziorek and Błażkiewicz, 2020). One such method is MSE, which quantifies the degree of balance between regularity and irregularity in the fluctuations of postural sway across multiple time scales, thereby capturing the complexity of the postural control system (Busa and van Emmerik, 2016). In the present study, MSE revealed reductions in sway complexity, a marker of diminished adaptability, among older adults with T2DM. Another widely used method is detrended fluctuation analysis (DFA), which quantifies the fractal scaling of postural sway. It yields a scaling exponent (α) that reflects long-range correlations, or self-similarity, in the data (Kantelhardt et al., 2001; Lin et al., 2008). For example, Koslucher et al. (2012) demonstrated greater predictability or self-similarity, as assessed by DFA, during the performance of a visual search task compared to the inspection (i.e., control) task in older adults. In our study, MSE provided complementary insights by revealing how adaptable or flexible the postural control system was across different time scales, which DFA does not address directly. Additionally, recent work has proposed a conceptual framework integrating complexity (MSE) and fractality (DFA) to track adaptive and maladaptive stages of physiological regulation during disease and aging (Busa et al., 2016). Within this framework, the onset of systemic constraints (e.g., T2DM) is marked by reductions in complexity, as measured by MSE. In parallel, fractal dynamics shift toward α ≈ 1.0, reflecting adaptive reorganization. As disease progresses, this adaptation breaks down: COP fluctuations lose scale invariance, α shifts away from 1.0, and MSE continues to decline. Together, these patterns indicate that both complexity and fractality are viewed as markers of maladaptive physiological states (Busa et al., 2016). Our findings suggest that MSE may be particularly sensitive for detecting early or subtle deficits in postural control among older adults with T2DM, providing information beyond what can be learned from DFA or other temporal dynamics measures alone.

Single-scale postural sway metrics were more effective than postural sway complexity measures in capturing both the group effect (i.e., T2DM) and the effect of task condition (i.e., control, visual search dual-task). These findings contrast with those of our previous study (Zhou et al., 2023), which suggested that while traditional sway measures, such as COP path length and sway area, can detect changes in postural control at a single scale, they may lack the sensitivity to capture intricate alterations in postural dynamics across multiple timescales, particularly those driven by subtle neurophysiological factors. Instead, sway complexity emerges as the method capable of capturing such nuanced variations in postural control. It is worth mentioning that in the study by Zhou et al. (2023), postural sway was quantified using COP measurements as participants stood on a Wii Balance Board. In the current study, we used a wearable accelerometer positioned near the center of mass, which has a higher test-retest reliability compared to COP measures (Whitney et al., 2011). Discrepancies in findings across studies may also be attributable to differences in participant population; for instance, Zhou et al. (2023) included individuals with cognitive impairment, a factor known to influence postural control, their sample consisted entirely of male participants. These differences raise the possibility that sex-related factors may have contributed to the observed variability in postural control outcomes. In contrast, the current study included both male and female participants, and we observed that females exhibited lower ML sway complexity across all conditions and groups. This reduced complexity may reflect less adaptable postural control strategies, which could help explain the higher incidence of falls and adverse events among females, as reported in previous studies (Kim et al., 2010; O’Neill et al., 1994; Stevens and Sogolow, 2005). Taken together, single-scale accelerometry and complexity sway metrics (multi-scale) may serve as complementary approaches for detecting alterations in postural control and identifying increased fall risk, particularly in vulnerable populations, including females.

Given the established correlation between postural adaptation and cognitive function (Yu et al., 2021), it is important to note that a decline in cognitive abilities can lead to reduced task performance and diminished postural control (Zhou et al., 2023). In the context of this relationship, our study also explored the connection between postural sway metrics (i.e., single-scale and multi-scale sway metrics) and their associated postural adaptations and visual search task performance within groups (i.e., non-diabetic, T2DM). In the current study, non-diabetic individuals who showed greater percent increases in elliptical sway area and range of acceleration from the control condition to the visual search condition–indicative of poorer postural adaptation–exhibited lower visual search accuracy. In other words, a lower percent change in postural sway metrics from control to dual-task reflects a more favorable response, indicating that participants were able to modulate or reduce their postural sway while performing the visual search task. These associations were not found in the diabetic group. These results are consistent with our prior work showing that within the non-dementia group, but not the dementia group, worse visual search task performance was associated with a greater increase in both ML and AP sway variability when engaged in the visual search task (as opposed to looking at a blank board) (Jor’dan et al., 2015). In this study, lack of association between postural adaptation and visual search task performance in the T2DM group suggests that the underlying mechanisms of diabetes may impact cognitive processes that are not readily detectable through global cognitive function assessments like the MMSE. This disruption in the synergy between perception and action underscores the need for future studies to utilize more comprehensive and precise cognitive assessment tools capable of identifying subtle cognitive changes associated with diabetes.

This study is the first to explicitly explore both single-scale sway metrics and postural sway complexity derived from accelerometry data during the performance of a visual search task in older adults with and without T2DM. With a relatively small sample size, we were able to detect group differences in single-scale metrics and ML sway complexity (before adjusting for covariates) during the performance of a visual search dual-task protocol. At the same time, this study had several limitations. Cognitive status was assessed using a relatively limited battery, which may not have been sensitive enough to detect subtle cognitive changes in individuals with T2DM. The cross-sectional design precludes causal inferences between T2DM, cognitive performance, and postural sway characteristics. Longitudinal studies are thus recommended to track changes in postural control over time and to better understand their relationship to fall risk in older adults with T2DM. Future research should recruit T2DM participants with varying levels of cognitive impairment and neuropathy severity, employ large sample sizes, utilize more comprehensive cognitive assessments, and examine the influence of different task constraints on postural sway complexity to provide deeper insight into the adaptability of the postural control system.

In conclusion, single-scale sway metrics in conjunction with the novel complexity metric based on accelerometry data may serve as cognitive-motor biomarkers to help predict and/or assess the loss of perception-action functionality in older adults with T2DM. In addition, future studies should focus on intervention strategies aimed at improving dual-task standing balance and preventing falls in older adults with T2DM.

## Data Availability

The raw data supporting the conclusions of this article will be made available by the authors, without undue reservation.
